# Autophagy in Human Skin Fibroblasts: Impact of Age

**DOI:** 10.3390/ijms19082254

**Published:** 2018-08-01

**Authors:** Hei Sung Kim, Seo-Yeon Park, Seok Hoon Moon, Jeong Deuk Lee, Sungjoo Kim

**Affiliations:** 1 Department of Dermatology, Incheon St. Mary’s Hospital, College of Medicine, The Catholic University of Korea, 222 Banpo-daero, Seocho-gu, Seoul 06591, Korea; hazelkimhoho@gmail.com (H.S.K.); windystep@naver.com (S.H.M.); leejd@olmh.cuk.ac.kr (J.D.L.); 2 Department of Medical Life Sciences, The Catholic University of Korea, 222 Banpo-daero, Seocho-gu, Seoul 06591, Korea; gkrud777@gmail.com

**Keywords:** autophagy, human skin fibroblasts, age, genetic analysis

## Abstract

Autophagy is an intracellular stress response that is enhanced under starvation conditions, and also when the cellular components are damaged. Aging accompanies an increase in intracellular stress and has significant impact on the skin. Since dermal fibroblasts are a powerful indicator of skin aging, we compared the autophagic activity of human skin fibroblasts between the young and old. According to TEM analyses, the number of autophagosomes per 1 μm^2^ cytoplasmic area was similar between young and aged fibroblasts. The amount of LC3 (microtubule-associated protein 1 light chain 3)-II, a form associated with autophagic vacuolar membranes, was also similar between the groups from Western blot analysis. Although residual bodies were more common in aged dermal fibroblasts, LC3 turnover and p62 assay showed little difference in the rate of lysosomal proteolysis between the young and old. RNA-seq analysis revealed that the major autophagy-modulating genes (*BECN1*, *MAP1LC3B*, *ATG5*, *ATG7*, *ULK1*, *PIK3C3*, *mTOR*) were not differentially expressed with age. Our results suggest that the basal autophagic flux in aged dermal fibroblasts is largely comparable to that of young fibroblasts. However, with a higher speed and amount of waste production in aged cells, we postulate that such autophagic flux may not be sufficient in keeping the old cells “clean”, resulting in skin aging. Aging is a complex process and, as such, the relationship between autophagy and aging is not straightforward. That is to say, autophagy does not simply decline with age. Regardless of the controversies on autophagic activity with age, autophagy plays a crucial role in counteracting aging, and strategies aimed at its modulation should hold promise for the prevention of skin aging.

## 1. Introduction

Aging results from an interaction of biological, physical and biochemical processes that cause cumulative damage to molecules and cellular function [1,2]. Autophagy is a finely-tuned “self-eating” process that disassembles unnecessary or dysfunctional components of the cell [3]. Constitutive (“house-keeping”) autophagy is important in clearing out damaged proteins or organelles, and adaptive autophagy is crucial in maintaining cellular homeostasis under starvation conditions or environmental stress [4]. Thus, autophagy is one of the survival mechanisms for cells against intrinsic and extrinsic stress and may be associated with age-related cellular dysfunction.

**Figure 1:**
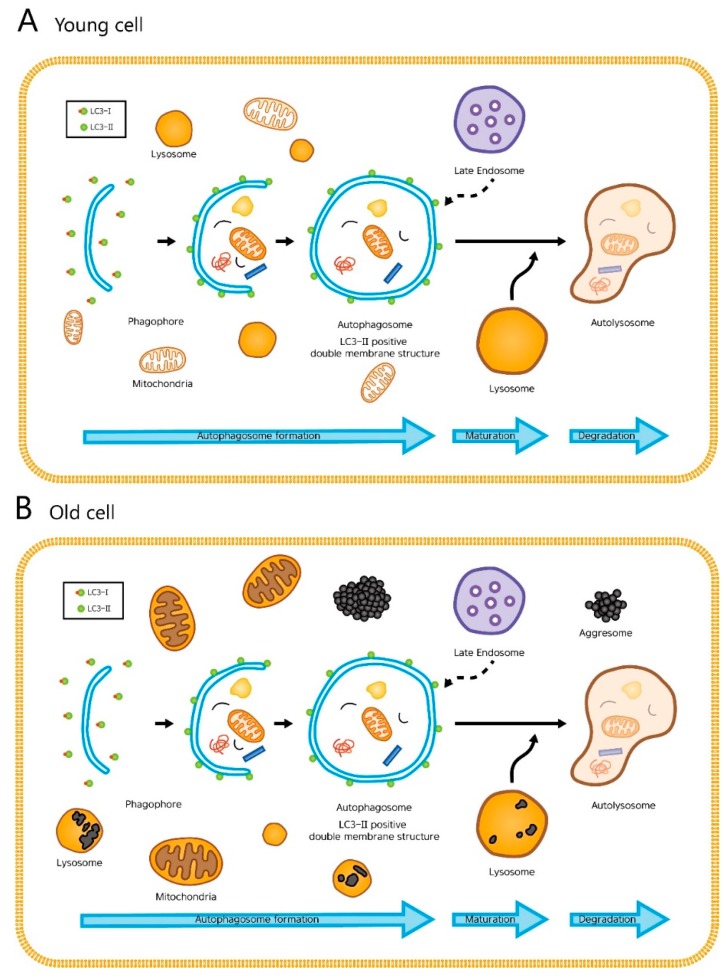
The cellular processes during autophagy. The whole dynamic process of autophagosome formation, autophagosome maturation, and autophagosome degradation is termed the “autophagic flux”. (**A**) In young cells, autophagy efficiently degrades damaged mitochondria or cytosolic proteins. (**B**) In old cells, lipofuscin occupies the lysosome, which hampers autophagy. Enlarged mitochondria and cytosolic proteins in indigestible aggregates (aggresomes) are increased.

Among the three types of autophagy (macroautophagy, microautophagy, and chaperone-mediated autophagy), macroautophagy (simply referred to as autophagy hereafter) is most extensively studied [5] and will be the focus of our paper. In autophagy, substrates (damaged organelles, cytosolic proteins and invasive microbes) are sequestered within double-membraned vesicles known as autophagosomes. The autophagosomes eventually fuse with lysosomes, resulting in degradation of their contents. This whole dynamic process of autophagy is termed the “autophagic flux” (Figure 1) [6].

Damaged proteins and organelles are known to accumulate in aged cells (Figure 1B). These changes result in a decrease of energy supply and an increase in intracellular damages and oxidative stress, all of which accelerates aging. With this said, it is easy to infer that gradual, age-related decline in autophagic activity contributes to the aging process. Not surprisingly, many have reported an age-related decrease in autophagic activity, especially autophagic degradation, which was determined by the levels of LC3-II in young and aged cells treated with chloroquine, an agent that impairs autophagic degradation by inhibiting lysosomal acidification and proteolysis [7,8,9,10]. Here, the aged cells had smaller increases in the level of LC3-II than young cells after chloroquine treatment, suggesting that the autophagic degradation is impaired in aged cells. However, others have reported no difference or even an increase in basal autophagic activity between the young and old, and the issue remains controversial [3,11].

Skin represents a valuable model to study aging in humans, since it is widely affected and is easily accessible. The characteristic signs of aged skin are wrinkles and skin sagging, which are caused by major changes in the dermal structure and a decrease in the collagen synthesis potential of dermal fibroblasts. With the importance of dermal fibroblasts in skin aging, the aim of this present study was to investigate the age-related changes of autophagy in human skin fibroblasts.

## 2. Results

### 2.1. Autophagosomes and Autolysosomes under Electron Microscopy

**Figure 2:**
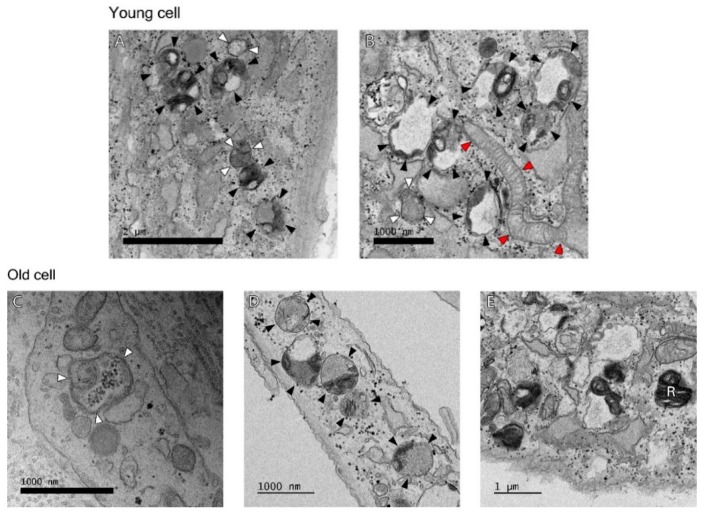
At the ultrastructural level, Young cell: (**A**) There are a number of autolysosomes (black arrowheads) at early stages of degradation. Different types of material are seen inside. There is already some degradation going on as the multi-membranous structure (called onion-like) is seen, which appears often when degradation is very active. An autophagosome with density similar to the surrounding cytosol and a double membrane (upper white arrowheads) and one with mitochondria inside (lower white arrowheads) are seen, (**B**) This is also an area of high autophagic activity where autophagosome (white arrowheads) and autolysosomes (black arrowheads) at different stages of degradation area seen. There is also a fresh, very long mitochondria fused to the mitochondrial network (red arrowheads). Old cell: (**C**) There is an autophagosome (white arrowheads) with a mitochondria inside. (**D**) A number of autolysosomes (black arrowheads) are seen. (**E**) A residual body (R), a lysosome containing indigestible materials is seen.

**Figure 3:**
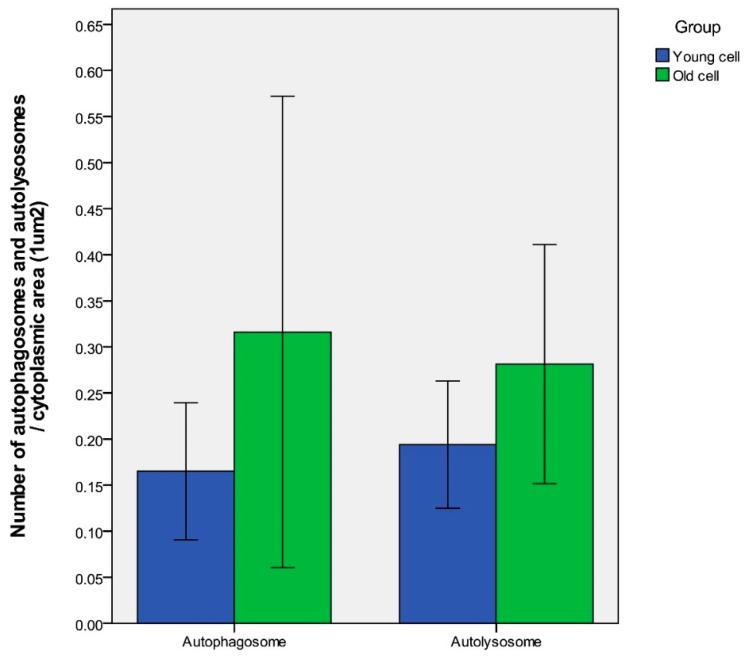
Under TEM, the number of autophagosomes per 1 μm^2^ cytoplasmic area were 0.17 ± 0.06 (young fibroblasts) and 0.32 ± 0.21 (aged fibroblasts) (*p* = 0.154). The number of autolysosomes per 1 μm^2^ cytoplasmic area were 0.19 ± 0.06 (young fibroblasts) and 0.28 ± 0.10 (aged fibroblasts) (*p* = 0.138). *n* = 5 in each group, and the numbers were counted in at least 12 cells. Data are provided as mean ± SD. *p* values < 0.05 were considered statistically significant.

Age-dependent differences of autophagy in dermal fibroblasts were examined by transmission electron microscopy (TEM). In the process of autophagy, significant amounts of cytosolic proteins and organelles are incorporated into autophagy-specific structures in the cytoplasm known as autophagosomes. At the ultrastructural level, an autophagosome is seen as a double-membraned structure containing undigested cytoplasmic contents. The autolysosome is a hybrid organelle generated by the fusion of an autophagosome and a lysosome. It has a single limiting membrane and contains cytoplasmic materials at various stages of degradation (Figure 2). The number of autophagosomes and autolysosomes in young fibroblasts (64 cells with a total area of 845.7 μm^2^) and aged fibroblasts (67 cells with a total area of 965.9 μm^2^) were counted under TEM. The number of autophagosomes per 1 μm^2^ cytoplasmic area were 0.17 ± 0.06 (young fibroblasts) and 0.32 ± 0.21 (aged fibroblasts) (*p* = 0.154). The number of autolysosomes per 1 μm^2^ cytoplasmic area were 0.19 ± 0.05 (young fibroblasts) and 0.28 ± 0.10 (aged fibroblasts) (*p* = 0.138) (Figure 3).

### 2.2. LC3-II Level and LC3-II Turnover Assay

**Figure 4:**
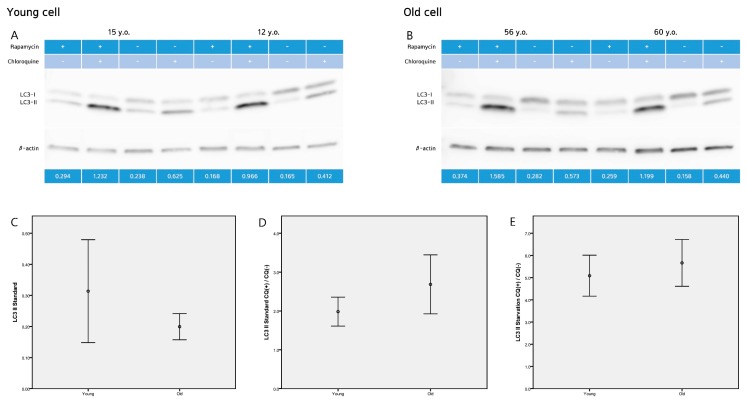
Western blot analysis (**A**,**B**) revealed that the level of LC3-II protein, were (**C**) 0.31 ± 0.23 (young fibroblasts) and 0.20 ± 0.76 (old fibroblasts) (*p* = 0.087) at standard (basal) conditions. (**D**) The ratio of LC3-II with and without chloroquine were 1.98 ± 0.52 (young fibroblasts) and 2.68 ± 1.37 (old fibroblasts) (*p* = 0.138) at standard conditions. (**E**) Under starvation conditions (induced by rapamycin), the ratio of LC3-II with and without chloroquine increased to 5.09 ± 0.52 (young fibroblasts) and 5.66 ± 1.90 (old fibroblasts) (*p* = 0.413). Images are representative of two separate experiments on *n* = 32 samples (15 young and 17 old). LC3-II expression is normalized to actin (arbitrary units). Data are provided as mean ± SD. *p* values < 0.05 were considered statistically significant.

Autophagosome formation takes conversion of cytoplasmic microtubule-associated protein 1 light chain 3 (LC3, mammalian ATG8) from a cytosolic (LC3-I) to a membrane-bound form (LC3-II) by adding phosphatidylethanolamine at its C-terminus. The amount of LC3-II closely correlates with the number of autophagosomes, and serves as a good indicator of autophagosome formation. Western blot analysis revealed that the level of LC3-II protein, were 0.31 ± 0.23 (young fibroblasts) and 0.20 ± 0.76 (old fibroblasts) (*p* = 0.087) at standard conditions (Figure 4).

One of the useful parameters to measure “autophagic flux” is the LC3 turnover, which is based on findings that LC3-II is degraded in autolysosomes. If cells are treated with lysosomotropic reagents such as chloroquine which raise lysosomal pH or inhibit autophagosome-lysosome fusion, the degradation of LC3-II is blocked, resulting in the accumulation of LC3-II. Accordingly, the differences in the amount of LC3-II between samples in the presence and absence of chloroquine denote the amount of LC3 that is delivered to the lysosomes for degradation.

We examined autophagic degradation by determining the levels of LC3-II in young and aged cells treated with chloroquine. Western blot analysis revealed that the ratio of LC3-II with and without chloroquine were 1.98 ± 0.52 (young fibroblasts) and 2.68 ± 1.37 (old fibroblasts) (*p* = 0.138) at standard conditions. Under starvation conditions (induced by rapamycin), the ratio of LC3-II with and without chloroquine increased to 5.09 ± 0.52 (young fibroblasts) and 5.66 ± 1.90 (old fibroblasts) (*p* = 0.413) (Figure 4).

### 2.3. p62 Assay

p62, also known as SQSTM1/sequestome 1, serves as a link between LC3 and ubiquitinated substrates, and is efficiently degraded by autophagy. Thus, the level of p62 protein can be used to monitor autophagic flux. The level of p62 protein by ELISA, were 11.3 ± 5.13 (young fibroblasts) and 13.4 ± 4.97 (old fibroblasts) (*p* = 0.293) at standard conditions. Under starvation conditions, p62 decreased to 10.7 ± 5.12 (young fibroblasts) and 13.1 ± 3.54 (old fibroblasts) (*p* = 0.162).

### 2.4. Gene Transcription Profile

**Figure 5:**
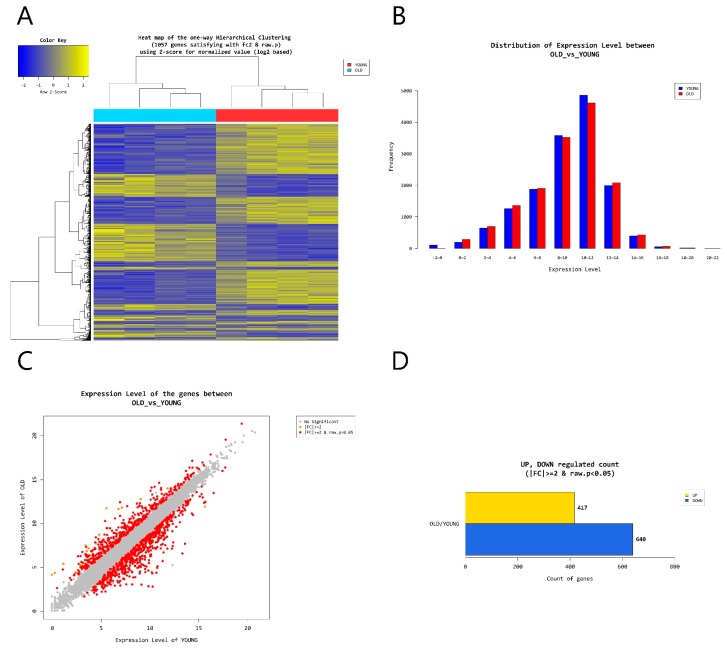
(**A**) Heat map of the one-way Hierarchical clustering. (**B**) Distribution of gene expression level between old and young dermal fibroblasts. (**C**) Scatter plot of gene expression level. (**D**) Significant gene count by fold change and *p*-value. *n* = 4 in each group. Only those genes exhibiting log2 fold change (FC) > 2 and *p* < 0.05 were considered to be differentially expressed genes.

According to RNA-Seq analysis, a total of 1057 genes were found to be differentially expressed by age (greater than 2-folds up and down and a raw *p*-value < 0.05). Among the 1057 genes, 417 genes were upregulated and 640 genes were downregulated in old cells (Figure 5). Supervised hierarchical cluster analysis illustrated on the heat map separates old fibroblasts and young fibroblasts into two distinct clusters (Figure 5). From gene-enrichment and functional annotation analysis using gene ontology, we identified that 4 autophagy-regulating genes (*FBXL2*, *HTR2B*, *MAPT*, *RAB33A*) were downregulated and 3 (*HK2*, *SOGA3*, *BNIP3*) upregulated in the old cells (*p* > 0.05) (Table 1).

**Figure 6:**
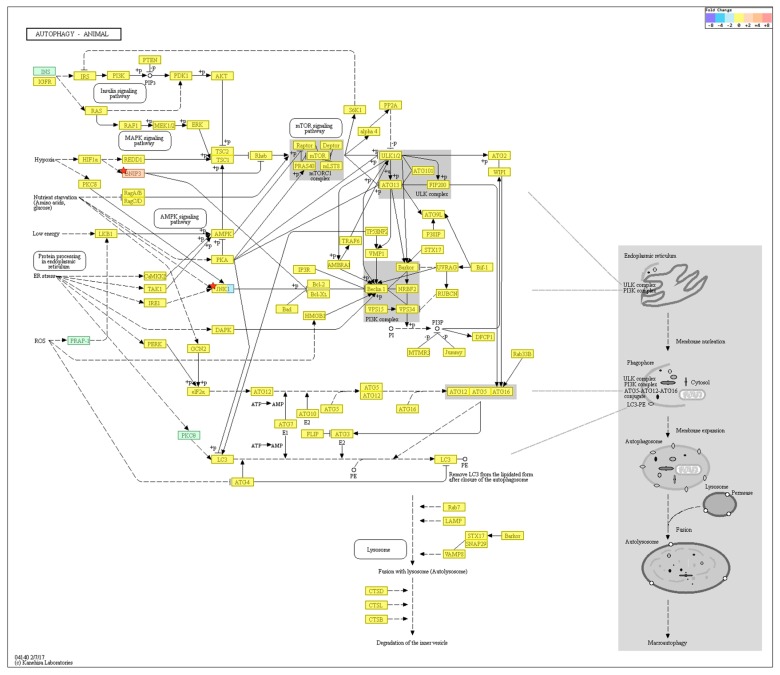
KEGG pathway analysis on autophagy. Among the genes, *BNIP3* was upregulated and *MAPK10* downregulated in the old cells but were not statistically significant. *n* = 4 in each group. Only those genes exhibiting log2 fold change (FC) > 2 and *p* < 0.05 were considered to be differentially expressed genes.

KEGG pathway analysis on autophagy is shown in Figure 6. Among the genes, *BNIP3* was upregulated (FC: 2.776557) and *MAPK10* downregulated (FC: −3.142697) in the old cells (*p* > 0.05).

## 3. Discussion

Aging is associated with progressive post-maturational deterioration of tissues and organs, which results in impairment of cell and tissue functioning, increased vulnerability to challenges and decreased ability of the organism to survive [12]. The aged population typically show accumulation of damaged proteins, which reflects an imbalance between the rates of protein damage and protein turnover [13,14]. Autophagy is a major proteolytic system [3,15] and thus impaired autophagy affects aging by reducing breakdown of altered proteins and by lengthening the “dwell time” of proteins in a cell, increasing their risk of becoming post-translationally altered [14,16,17,18].

There is evidence that the decline in the ability of the lysosomes to degrade intracellular components (later stage of autophagy) may be the major cause of reduced protein breakdown in aging. The presence of undigested materials in the lysosomes (residual bodies or lipofuscin), as seen in our old fibroblasts (Figure 2E), can be responsible for their impaired ability to fuse and/or to degrade the autophagosome contents. Recent studies also point towards defective autophagosome formation to participate in the aging process [18,19,20,21].

Skin aging is a cumulative process that affects skin function and appearance. Despite the interest in age-dependent skin changes, the role of autophagy in chronological skin aging has not been widely examined. To better understand the mechanism of skin aging, our study focused on age-related changes of autophagy in cells which constitute the dermis. From TEM, we found the number of autophagosomes per 1 μm^2^ cytoplasmic area to be similar between aged dermal fibroblasts (fibroblasts derived from the skin of elderly men) and young dermal fibroblasts (fibroblasts derived from the skin of young boys). In addition, LC3-II levels of the old and young fibroblasts were also statistically similar, which suggest that autophagosome formation in aged dermal fibroblasts is no less than to that of young fibroblasts. As for lysosomal degradation, the number of autolysosomes/1 μm^2^ and the rate of lysosomal proteolysis (monitored by LC3 turnover assay and p62 assay) in old fibroblasts were not statistically different from that of young fibroblasts. In terms of gene-enrichment and functional annotation analysis using gene ontology, we identified a two-fold change in expression of seven autophagy-regulating genes (Table 1) in old cells, but none were statistically significant. As for KEGG pathway analysis, the expression of top-ranked autophagy regulators (*BECN1*, *MAP1LC3B*, *ATG5*, *ATG7*, *ULK1*, *PIK3C3*, *mTOR*) [22] were not drastically altered. A two-fold change in expression was seen with BNIP3 and MAPK10, but they too were not statistically significant.

**Figure 7:**
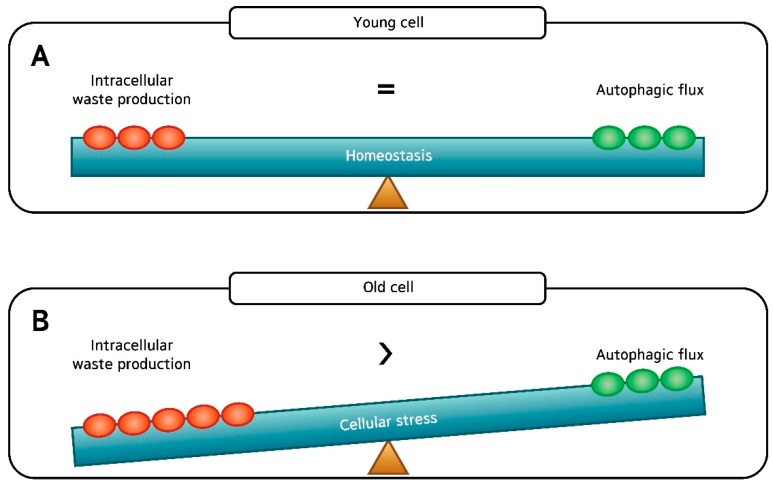
(**A**) There is good balance between “intracellular waste production” and “autophagic flux” in young dermal fibroblasts; and (**B**) Despite the apparently intact autophagic flux, it is likely that the rate of “intracellular waste production” exceeds the rate of autophagic flux (“waste removal”) in old fibroblasts, which may accelerate skin aging.

Collectively, our results suggest that basal autophagic flux in aged dermal fibroblasts is comparable to that of young fibroblasts. Although our findings are contrary to the dogma that autophagic activity declines with age, we believe that we assessed the autophagic flux as strictly as possible. Our finding is in line with the observations that have been made by Demirovic et al. [3], who reported no age-related difference of LC3 fluorescence in full-thickness skin sections. Yamamoto et al. [11] even demonstrated substantial upregulation of autophagy in the aged kidney where they speculate that age-related increase in basal autophagic activity is compensatory (that is, to cope with time-dependent accumulation of cellular “garbage”). We too expect increased damage to the cellular components in aged cells compared to their young counterpart. With a higher speed and amount of waste production in aged cells, autophagic flux that is ample for a young cell may not be sufficient in keeping the old cells “clean”. To summarize, despite the apparently intact autophagic flux found in our study, it is likely that the rate of “intracellular waste production” exceeds the rate of autophagic flux (“waste removal”) in old fibroblasts, resulting in skin aging (Figure 7).

Caloric restriction is known to prevent the accumulation of altered proteins and the age-related dysfunction of autophagic proteolysis. Additionally, in principle, long-lasting administration of caloric restriction mimetics and stimulators of macroatuophagy via mTOR blockage like rapamycin, should have similar effects [23]. The concept was backed up by our study results where the addition of rapamycin dramatically increased the rate of autophagic degradation in both the aged and young fibroblasts.

As mentioned earlier on, the role of autophagy in skin aging is yet poorly understood, especially in the dermis. We have tried to overcome several drawbacks present in prior studies and believe that our study is perhaps the largest (*n* = 32, 15 young and 17 old) with a wide age-gap between the young and old cells (mean age 12.7 vs. 67) to date, that compares basal autophagic flux in human dermal fibroblasts with age.

We have adopted the currently available techniques and methods to monitor and modulate autophagy in human cells [24,25,26,27,28]. As for monitoring the number of autophagosomes, a count was performed under electron microscopy, which is the most traditional method. Biochemical detection of the membrane-associated form of LC3 (LC3-II) was also performed. One critical point that must be kept in mind is that autophagy is a highly dynamic, multi-step process. The accumulation of autophagosomes can indicate either autophagic activation or a blockage of downstream steps in autophagy, such as inefficient fusion or decreased lysosomal degradation (Figure 1). Since electron microscopy and LC3-II measurement do not provide direct information about lysosomal degradation of autophagic substrates, it is not classified as a formal “autophagic flux” assay. To overcome this problem, we monitored LC3 turnover and analyzed gene expression (including functional analysis and KEGG pathway analysis for autophagy-regulating genes) with age.

We emphasize that there is currently no “gold standard” to monitor or modulate autophagic activity. As we further clarify the molecular mechanisms of autophagy, better assays and agents will be developed to monitor and modulate autophagy. Aging is a complex process and as such, the relationship between autophagy and aging is not straightforward. That is to say, autophagy does not simply decline with age. Regardless of the controversies on autophagic activity with age, autophagy plays a crucial role in counteracting aging, and strategies aimed at its modulation should hold promise for the prevention of skin aging.

## 4. Materials and Methods

### 4.1. Skin Samples

Fifteen foreskin samples (ages 9–18 years old, average 12.7) and 17 truncal skin tissues (ages 50–94 years old, average 67) were collected from healthy Korean males within the months of February to May. None of the donors had any medical condition or was under medication.

### 4.2. Ethics

This study was approved by the institutional review board of Incheon St. Mary’s Hospital, The Catholic University of Korea (OIRB-00252-004, (25 January 2016)). Eligible patients/guardians were informed about the study protocol in clear, simple language before an informed consent was obtained.

### 4.3. Cell Culture

Human dermal fibroblasts were isolated from the tissue specimens by enzymatic digestion using collagenase (500 U/mL; GIBCO, Grand Island, NY, USA). Cells were cultured in Dulbecco’s Modified Eagle’s Media-high glucose (DMEM; Sigma-Aldrich, St. Louis, MO, USA) supplemented with 10% fetal bovine serum and 1% penicillin/streptomycin at 37 °C in a 5% CO_2_ humidified incubator. All cells used in the present study were obtained from the second cell passage. A proportion of cells were treated with rapamycin (30 μM; Sigma) for 24 h and/or chloroquine (50 μM; Sigma) for 4 h before cell preparation.

### 4.4. Cell Lysis and Western Blotting

Cell pellets were dissolved in RIPA (Radioimmunoprecipitation assay) lysis buffer (1% NP40, 0.5% Na-DOC, 0.1% SDS, 50 mM Tris-HCl with pH 8.8) containing Protease Inhibitor Cocktail (genDEPOT, Houston, TX, USA). Protein concentration was measured using the Bradford assay (Bio-Rad Laboratories, Hercules, CA, USA, 5000006). The separated proteins were subsequently transferred to a nitrocellulose membrane and were subjected to immunoblotting with antibodies specific for LC3B (abcam, Cambridge, UK, ab51520) and β-actin (abm, Vancouver, BC, Canada, G043). Signal was detected with appropriate HRP (horseradish peroxidase) conjugated secondary antibodies and an ECL system (Dong-In Biotech, Seoul, Korea). Western blot films were scanned with a Cannon Flatbed scanner and analyzed using the ImageJ gel analysis software (Version 1.44; NIH, Bethesda, MD, USA). Band intensities were quantified and made reference to actin control bands. The Western blot was performed on all 32 samples, twice as independent experiments, with cells used at the same passage number.

### 4.5. Enzyme-Linked Immunosorbent Assay (ELISA)

The cells and supernatant were collected from the primary fibroblast culture and protein expression of p62 was evaluated by ELISA (Enzo Life Sciences, Farmingdale, NY, USA, ADI-900-212-0001). Briefly, each well was blocked with blocking buffer for 2 h and washed with wash buffer. Antibody against p62 was added to the media and incubated for 2 h. A substrate solution and stop solution was introduced sequentially and the optimal density of each well was determined within 30 min using a microplate reader (Bio-Rad). All 32 samples were analyzed in duplicate (replicate wells).

### 4.6. Transmission Electron Microscopy (TEM)

Cells were fixed with 2.5% glutaraldehyde in 0.1 M PBS after harvesting overnight at 4 °C and washed with 0.1 M PBS. After immersion in 2% agarose gel, the cells were post-fixed in 4% osmium tetroxide solution for 1 h. After washing with distilled water, the cells were dehydrated in a graded series of ethanol (30%, 60%, 70%, 90%, and 100%), stained with 0.5% uranyl acetate (1 h), and finally embedded in epoxy resin. The resin was polymerized at 60 °C for 48 h. Ultrathin sections were prepared with a ultramicrotome and then stained with 5% aqueous uranyl acetate and 2% aqueous lead citrate. After air drying, the sections were observed on TEM (JEOL, Tokyo, Japan, JEM-2100). A total of 10 samples (5 young and 5 old) were examined.

### 4.7. mRNA-Seq

In constructing cDNA libraries with the TruSeq RNA library kit, 1 μg of total RNA was used. The protocol consisted of polyA-selected RNA extraction, RNA fragmentation, random hexamer primed reverse transcription and 100nt paired-end sequencing by Illumina HiSeq4000. The libraries were quantified with qPCR based on the qPCR Quantification Protocol Guide and qualified using an Agilent Technologies 2100 Bioanalyzer.

We preprocessed the raw reads and aligned the processed reads to the *Homo sapiens* (*hg19*) using HISAT v2.0.5 [26]. HISAT utilizes two types of indexes for alignment (a global, whole-genome index and tens of thousands of small local indexes) and generates spliced alignments that is several times faster than Bowtie and BWA (Burrows-Wheeler Aligner). The reference genome sequence for *Homo sapiens* (*hg19*) and the annotation data were downloaded from the UCSC genome browser database (http://genome.uscs.edu). After alignment, StringTie v1.3.3b (http://ccb.jhu.edu/software/stringtie/) was used to assemble aligned reads into transcripts and to estimate their abundance as FPKM values (Fragments Per Kilobase of exon per Million fragments mapped) [27,28]. FPKM values are normalized with respect to library size and are used for comparison analysis of differentially expressed genes between samples. A total of 8 samples (4 young and 4 old) were examined.

### 4.8. Statistical Analysis

Difference between the young and aged cell group was analyzed using the unpaired *t*-test. *p* values < 0.05 were considered statistically significant. Statistical analysis of data was performed using SPSS for Windows software (version 15.0; SPSS Inc., Chicago, IL, USA)

Differentially expressed genes were analyzed using the estimates of abundances for each gene in samples. Genes with at least one zero FPKM value in the samples were excluded. To facilitate log2 transformation, 1 was added to each FPKM value of filtered genes. Filtered data were log2-transformed and subjected to quantile normalization. Statistical significance of the differential expression data was determined using independent *t*-test and fold change in which the null hypothesis was that no difference exists among groups. False discovery rate (FDR) was controlled by adjusting *p*-value using the Benjamini–Hochberg algorithm. For DEG (Differentially Expressed Gene) set, hierarchical clustering analysis was performed using complete linkage and Euclidean distance as a measure of similarity. Gene-enrichment and functional annotation analysis and pathway analysis for significant gene list were performed based on Gene Ontology (www.geneontology.org/) and KEGG pathway (http://www.genome.jp/kegg/pathway.html).

## Figures and Tables

**Table 1:** From functional analysis using gene ontology, 4 autophagy-regulating genes (*FBXL2*, *HTR2B*, *MAPT*, *RAB33A*) were found downregulated and 3 (*HK2*, *SOGA3*, *BNIP3*) upregulated in the old cells compared to their young counterpart (*p* > 0.05).

**Autophagy-Regulating Genes Downregulated in the Old Cells**		
	OLD/YOUNG. fc	*p*-value
*FBXL2*	−4.021224	>0.05
*HTR2B*	−2.349009	>0.05
*MAPT*	−4.279171	>0.05
*RAB33A*	−5.610289	>0.05
**Autophagy-Regulating Genes Upregulated in the Old Cells**		
	OLD/YOUNG. fc	*p*-value
*HK2*	2.100391	>0.05
*SOGA3*	2.114569	>0.05
*BNIP3*	2.776557	>0.05

## Abbreviations

TEM	Transmission electron microscopy
LC3	Microtubule-associated protein 1 light chain 3
BECN1	Beclin-1
MAP1L3B	Microtubule-associated protein 1A/1B light chain 3B
Atg5	Autophagy related 5
Atg7	Autophagy related 7
ULK1	Serine/threonine-protein kinase
PIK3C3	Phosphatidylinositol 3-kinase catalytic subunit type 3
mTOR	Mammalian target of rapamycin
FBXL2	F-box and leucine rich repeat protein 2
HTR2B	5-hydroxytryptamine receptor 2B
MAPT	Microtubule-associated protein Tau
RAB33A	Member RAS oncogene family
SOGA3	Suppressor of glucose, autophagy associated 3
BNIP3	Bcl2/adenovirus E1B 19-kDa interacting protein 3
